# Comparison of Patterns of Use of Unrecorded and Recorded Spirits: Survey of Adult Drinkers in Rural Central China

**DOI:** 10.3390/ijerph14101099

**Published:** 2017-09-22

**Authors:** Shiqing Wei, Ping Yin, Ian M. Newman, Ling Qian, Duane F. Shell, Lok-wa Yuen

**Affiliations:** 1 Department of Epidemiology and Health Statistics, School of Public Health, Tongji Medical College, Huazhong University of Science and Technology, Wuhan 430073, China; flowerfir@outlook.com; 2 Department of Educational Psychology, University of Nebraska-Lincoln, Lincoln, NE 68588, USA; dshell2@unl.edu (D.F.S.); lok-wa.yuen@huskers.unl.edu (L.Y.); 3 Chinese Center for Health Education (CCHE), Beijing 100011, China; qianlingzh@126.com

**Keywords:** grain spirits, distilled spirits, bai jiu, noncommercial alcohol, unrecorded alcohol, drinking patterns, alcohol preferences, gender differences

## Abstract

About 70% of the beverage alcohol consumed in China annually is spirits. Recorded spirits make up most spirit consumption, but about 25% of total alcohol consumption (1.7 L pure alcohol per capita annually) is unrecorded spirits (bai jiu), either homemade or made in unregulated distilleries. In some parts of China, the consumption of unrecorded spirits is higher than average. This paper compares the patterns of use of unrecorded distilled spirits and recorded distilled spirits among rural residents in Central China. Interviews were conducted with 3298 individuals in 21 towns/villages in 10 counties in the Hubei, Anhui, and Hebei provinces in the People’s Republic of China. Unrecorded bai jiu drinkers chose it because of its taste and its low price. It was consumed mostly by older men, mostly at home with family, more regularly and at higher alcohol by volume (ABV) compared to recorded alcohol. Recorded bai jiu drinkers were more likely to drink away from their homes, consumed more bai jiu at memorable drinking occasions, and reported feeling sick after drinking more often than unrecorded bai jiu drinkers. This comparison of patterns of use of unrecorded bai jiu and recorded bai jiu does not suggest that unrecorded bai jiu is more problematic for drinkers.

## 1. Introduction

The Chinese public does not generally consider alcohol use a significant public health problem. Rather, drinking alcohol is considered a normal part of daily life, a usual part of many meals, an important part of medicine and religion, and an essential adjunct for celebrations, ceremonies, and business [1]. This benign view is being challenged by the rising consumption of alcohol. The World Health Organization (WHO) most recently estimated the average 2008–2010 per-capita alcohol consumption for China at 6.7 L of pure alcohol, up 37% from a 4.9 L average for 2003–2005 [2].

Current estimates are that 70% of the alcohol consumed in China is spirits, and 25% of total alcohol consumption (1.7 L pure alcohol per capita annually) is unrecorded spirits (bai jiu), either homemade or made in unregulated distilleries [2]. In rural areas, the percentage for unrecorded alcohol could be much higher [3,4]. Unrecorded distilled grain spirits (bai jiu) are legally produced on a small scale throughout rural China for family use and to be traded and/or sold on a limited scale to neighbors and local markets. Recorded bai jiu is produced and sold by licensed distilleries. A recent review of literature on unrecorded alcohol acknowledged that there is a “dearth of information” about unrecorded alcohol in China [5]. Unrecorded alcohol risks to public health have been highlighted by the alcohol industry [6]. The industry notes risks related to contamination and adulteration, the market disruption from counterfeiting, and the unfairness of having a marketplace competitor that is untaxed and mostly unregulated in its production, distribution, and sale. Problems of unrecorded alcohol have been reported in Eastern Europe, Central and South America, and Africa, and in Asia (mostly from the Indian subcontinent) [6]. One field study described China’s unrecorded distilled spirits production [3], and three papers have described chemical analysis of Chinese unrecorded distilled spirits samples [7,8,9]. These few China studies, so far, found no unusual problems arising from production of unrecorded distilled alcohol. This study looked at individuals’ patterns of use of unrecorded spirits to see if it differed markedly from the use of recorded spirits and if any pattern of use of unrecorded spirits warrants further study.

Bai jiu, which literally means “white spirits”, was selected for study because (1) it is the type of alcohol that has been traditionally preferred by the majority of Chinese drinkers; and (2) the higher ABV (alcohol content by volume) poses potentially more health risks to drinkers compared to lower-ABV beverages such as beer or wine. Chinese-style distilled spirits are readily available for sale everywhere, in the form of commercial brands (regulated, taxed) and unrecorded spirits (unregulated–casually regulated, not taxed). It is legal to make, sell, purchase, and consume unrecorded spirits. This paper uses the terms bai jiu and spirits interchangeably.

This study was designed to gather information on individual-level patterns of use of unrecorded and recorded spirits in rural Central China. This paper is the first to begin to offer basic information on patterns of use.

## 2. Materials and Methods

### 2.1. The Setting

Three provinces in Central China were selected for study—Anhui, Hebei, and Hubei—based on common knowledge of widespread use of unrecorded bai jiu in the rural areas.

### 2.2. Sampling

The sampling plan was designed to identify and interview bai jiu drinkers, especially unrecorded bai jiu drinkers and was not designed to be demographically representative of bai jiu use. Multistage sampling was used. In Anhui and Hebei, four counties were selected and then two townships/villages were selected within each county. In Hubei, five towns in two counties were selected. Households within townships/villages were then selected for interviews. Eligible household residents were 18 years and older who had consumed alcohol in the last year. Interviewers were instructed to interview the oldest male, the youngest male over age 15, and one female in the household during the interview period. The male–female ratio reflects the approximate proportion of male and female drinkers in the adult population [2,4,10].

The final sample was from 21 towns/villages in 10 counties in 3 provinces. A total of 3298 individuals were interviewed, and 3268 interviews were successfully completed (99.1%); Hubei *n* = 1110; Anhui *n* = 1070; Hebei *n* = 1088).

The classification of bai jiu drinkers into unrecorded and recorded bai jiu drinkers was based on the answer to the question, “What type of alcohol do you usually drink?” Of the 3048 bai jiu drinkers, 1722 (56.5%) usually drank unrecorded bai jiu and 1326 (43.5%) usually drank recorded bai jiu.

Of the 3268 people interviewed, 3048 (93.2%) were self-reported bai jiu drinkers: 84.2% males and 15.1% females. The mean age of males and females was essentially the same: males: 49.1 years; females: 49.8 years. Occupations were typical for people in rural communities: 52.6% were engaged in crop farming, 9.4% in service industries, 6.4% in construction, 5.0% in livestock farming, 4.5% in transportation, and 22.2% in other occupations including factory work, local government employee, unemployed, and house wife/mother. Most of the participants (91.9%) were married, a majority (77.8%) had completed middle school or less, and about one-fourth (22.1%) had attended high school or technical school of some type.

### 2.3. Interview Strategy

Data were gathered by interview rather than written questionnaire because of the range of literacy among the intended sample and because of the need to clearly explain the definition of alcohol use. Because alcohol tends to be regarded as a banal part of daily diet and of socializing its casual use is often overlooked. For example, to a general question such as “do you drink alcohol?” a person may only mention drinking that is associated with special celebrations and events. Interviewers made clear that people should count all alcohol use, even alcohol taken with meals and alcohol taken as medicine.

Before an interview began, interviewers explained the purpose of the interview, and assured participants that they would remain anonymous, could decline to answer any questions, and could leave the interview at any time without any negative consequences. All those interviewed signed a consent form. University graduate students studying epidemiology and public health gathered the interview data after being trained on the study protocols. Each interview took approximately 15 min. The Institutional Review Board for the Use of Human Subjects at the University of Nebraska—Lincoln approved the project (Approval #: 20120412640 EX). Additional approvals were obtained from Tongji Medical College and from the provincial authorities.

### 2.4. Questionnaire Development

A working group of senior Chinese provincial public health officials, Chinese epidemiologists, and United States university epidemiologists and social scientists developed and pretested the questionnaire. The initial framework for the questionnaire was based on previous research in China [11,12,13,14,15,16,17,18,19] and used commonly accepted terms to ensure comprehension. For example, questions about quantity of spirits consumed were asked in terms of liang, because liang is the most widely used and understood measure of alcohol quantity in this part of China. One liang is approximately equal to 50 g.

### 2.5. Analysis

Data from the interviews were transferred to computer files at Tongi Medical College of Huazhong University of Science and Technology, Wuhan, China, using Epidata 3.0 (http://www.epidata.dk/cn/index.htm) for data entry. Demographic information and data about drinking behaviors, choice of bai jiu, drinking experience, and consequences were analyzed using SAS 9.2 (SAS Institute Inc., Cary, NC, USA).

## 3. Results

Table 1 presents the demographic characteristics for the drinkers of the two types of bai jiu. The majority of the male bai jiu drinkers (57.7%) consumed unrecorded bai jiu. Among the female bai jiu drinkers, the proportion of drinkers of unrecorded and unrecorded bai jiu drinkers was essentially equal: 49.7% and 50.3%. The proportion of males drinking unrecorded bai jiu increased with age; this was not so among the females.

### 3.1. Drinking Frequency

Information about drinking frequency is shown in Table 2. Unrecorded bai jiu drinkers drank more frequently (3 days or more per week) than the recorded bai jiu drinkers. Unrecorded bai jiu drinkers were more likely to drink higher strength bai jiu (42% and higher alcohol by volume) than were recorded bai jiu drinkers.

### 3.2. Estimated Quantity

The quantity of bai jiu consumed on a “usual” drinking occasion and the “last” drinking occasion were essentially the same for both groups. Unrecorded bai jiu drinkers reported drinking significantly less than recorded bai jiu drinkers at their “most memorable” drinking occasion (Table 3).

### 3.3. Reasons for Drinking Unrecorded Spirits

Unrecorded bai jiu drinkers selected unrecorded bai jiu as their usual drink because it was cheap (35.9%), it tastes good (27.1%), it was provided by host or as a gift (13.4%), it was easy to get (7.0%), it was good for health (8.3%), and it was at good quality (5.8%). Unfortunately, we did not ask recorded alcohol drinkers why they selected recorded alcohol.

### 3.4. Patterns: Where and with Whom and Consequences

On their “last drinking occasion”, unrecorded bai jiu drinkers were most likely to be drinking in their own home (61.3%) with family members (32.4%); the next most likely place was the home of a relative or friend (16.6%) or in the company of relatives or friends (22.8%). Recorded bai jiu drinkers were slightly less likely to be drinking in their own homes (50.1%), less likely to be drinking with family members (24.3%), and more likely to be drinking in the home of a friend or a relative (30.5%) or in the company of friends or relatives (40.4%).

On the “most memorable drinking occasion”, only 38.7% of unrecorded bai jiu drinkers were drinking at their own home or in the home of a friend or relative (20.8%). Recorded bai jiu drinkers were less likely to be drinking at their own home (26.5%) than in the home of a friend or relative (33.3%) and much more likely to be drinking in the company of friends and relatives (52.9%) than in the company of family members (18.1%).

To gain some insight into the stability of drinkers’ preferences, we asked the bai jiu drinkers what they chose to drink at their last drinking occasion and their most memorable drinking occasion. There were differences between the two groups in the type of bai jiu they selected for different occasions (Table 4). The proportion of unrecorded bai jiu drinkers who drank their usual type of spirits was lower on their most memorable drinking occasion compared to their last drinking occasion. The proportion of recorded bai jiu drinkers who drank recorded bai jiu on these occasions remained consistently high. Given a choice, 34.1% of the unrecorded bai jiu drinkers said they would prefer to drink recorded bai jiu.

There were small but not meaningful differences between the proportion of the two groups who pressured others to drink or were themselves pressured to drink by others in the traditional Chinese custom of toasting. Half of the groups experienced being pressured to drink, and nearly two-fifths of the groups had pressured others to drink. Drinkers of unrecorded bai jiu were, however, significantly less likely to report feeling sick or vomiting as a result of drinking than were drinkers of recorded bai jiu (Table 5).

## 4. Discussion

To be eligible for the interview in this study, a person had to have drunk alcohol in the past year; thus, all (100%) of the persons in this study were drinkers. An estimate of the drinking rates was not the purpose of this study. In this sample of rural drinkers, only 6.7% had not or did not drink bai jiu, preferring to drink beer or wine exclusively. More than half of the bai jiu drinkers (56.5%) drank unrecorded spirits as their usual choice of alcohol. WHO’s national estimated average for unrecorded alcohol use in China was 25% [2], but our finding is similar to the results for studies in rural areas that tabulate rural and urban drinking separately [3,4], suggesting comparability.

This study found that the main differences in patterns of use between people who preferred unrecorded bai jiu compared to those who preferred recorded bai jiu were (1) an age-related preference for unrecorded spirits among older men, (2) a preference for higher-strength bai jiu among unrecorded bai jiu drinkers, (3) a greater likelihood of daily drinking among unrecorded bai jiu drinkers, (4) a greater likelihood of drinking at home on the last drinking occasion and the most memorable occasion among the unrecorded bai jiu drinkers, (5) a tendency for unrecorded bai jiu drinkers to cross over to drinking recorded bai jiu at opportunities such as memorable occasions, (6) recorded bai jiu drinkers reported heavier drinking on memorable drinking occasions than unrecorded bai jiu drinkers, and (7) more of the recorded bai jiu drinkers reported feeling sick or vomiting after drinking.

Male drinkers were more likely to consume unrecorded bai jiu as their usual drink, and older men showed a preference for unrecorded bai jiu. Traditionally in China drinking for females has been restricted to situations like being offered a drink as a sign of hospitality, festival-related drinking, and special occasion drinking. Females are typically passive drinkers who exercise little choice over the type of alcohol they drink. In contrast, males are actively hosting drinking occasions, using alcohol to celebrate social occasions, and using alcohol to initiate and reinforce social relationships.

Unrecorded bai jiu drinkers were more likely to drink daily or more frequently during the week than recorded bai jiu drinkers, and they preferred higher ABV bai jiu. This is likely a reflection of long-standing practice and traditional drinking patterns. Alcohol in these settings is considered an unremarkable beverage that is usually served with meals and for refreshment. Many rural people believe there is a health benefit from the moderate consumption of spirits, and, while acknowledged as an intoxicant, the motivation for drinking is related to alcohol’s properties as a food more than as a mood modifier. The preference for higher ABV beverages may be linked to a tradition of admiring men who drink stronger alcohol [1,20]. How much a pattern of daily drinking and drinking higher strength bai jiu contributes to the alcohol-related burden of disease is unknown. On one hand, as long as alcohol use is not excessive, then drinking with meals and spacing drinks throughout the day is a lower risk pattern compared to drinking the same quantity of alcohol in a single sitting [21,22]. On the other hand, it has been estimated that 50% of Asians inherited a genetic trait that interferes with metabolization of ethanol. This genetic trait is most recognizable in alcohol-related facial flushing. This condition increases risk for some aerodigestive cancers [23,24]. A pattern of daily use and a preference for higher strength spirits may present a higher risk for these individuals [25,26].

We attempted to assess the quantity of bai jiu consumed. The average quantity reported per usual drinking occasion was 2.7–2.9 liang. There are several reasons for caution when interpreting self-reported alcohol quantity data from China due to a number of difficult-to-control factors that detract from accuracy [11,12,13,14,15,16,17,18,19]. For example, alcohol is purchased in containers of different sizes, there is no standard serving size or serving glass, and spirits of different strengths (ranging from 30% to 60% ABV) are mixed together when guests’ drinks are “topped up” by their friends or host in social drinking situations. Interestingly, during fieldwork, we observed that unrecorded bai jiu drinkers, especially the older drinkers, estimated liang (quantity) more accurately than recorded bai jiu drinkers and younger drinkers. We think this is because the unrecorded bai jiu drinkers tend to drink consistently one type/strength of bai jiu in situations not involving active social drinking (at home) so their estimates are likely to be more accurate.

One of the reasons given by respondents for choosing unrecorded bai jiu is that it is cheap. Low cost unrecorded alcohol has been suggested to increase burden of alcohol-related diseases because it potentially enables excessive use [5]. However, these data did not support low cost driving up bai jiu use, nor did a 2011 analysis of national data from 1993–2006, which reported almost no effect of price on alcohol consumption in China [27]. Our data showed that only on “memorable” drinking occasions does the quantity of bai jiu consumed differ. On “memorable” occasions, it was the recorded bai jiu drinkers, consuming the more expensive product, who reported higher use. This difference in drinking pattern appears to be the result of the social circumstances associated with “memorable” drinking occasions.

Bai jiu plays a traditional role in festivities, especially in rural China. Spring and Fall Festivals, as well as other traditional feast days, funerals, weddings, and village events are celebrated with toasting and companionable social drinking. These are occasions on which drinking etiquette might allow a greater margin for heavy drinking and some boisterous behavior. Both unrecorded and recorded bai jiu drinkers reported higher alcohol consumption on their “most memorable” drinking occasion, but recorded bai jiu drinkers drank more than the unrecorded bai jiu drinkers.

This increased consumption by recorded bai jiu drinkers on “memorable” drinking occasions perhaps explains why they were more likely to report that they “felt sick or vomited after drinking” (a proxy measure for heavy drinking). However, because the unrecorded bai jiu drinkers tended to be older and to drink regularly, they may have developed a degree of tolerance and/or skill in pacing their drinking; therefore, feeling sick or vomiting after drinking may not be strongly associated with heavy episodic drinking. Nevertheless, our data showed that even on their “most memorable” drinking occasions the unrecorded bai jiu drinkers consumed fewer drinks than the recorded bai jiu drinkers.

Certain customs that apply to group drinking in China can increase risk of heavy episodic drinking and intoxication: toasting, keeping up with others, topping up drinks, drinking games, and competitions. Drinkers in this study were asked if they had pressured others to drink and if they had been pressured to drink by others. Tellingly, there was no difference between unrecorded bai jiu drinkers and recorded bai jiu drinkers, suggesting that these drinking patterns were independent of the type of spirits involved and likely deeply grounded in Chinese drinking culture.

The choice of drinking location could affect the potential risk of unintentional injury. Unrecorded bai jiu drinkers preferred to drink at their own home with family, while the recorded bai jiu drinkers were more likely to drink at the home of friends or relatives. This difference was most evident for “memorable” drinking occasions. In addition to the potential family controls associated with drinking at one’s own home compared to drinking away from home is added the consideration that drinking away from home may involve driving after drinking.

### Policy Implications for Unrecorded Spirits

Some of the policy recommendations outlined in the WHO’s *Global Strategy to Reduce Harmful Use of Alcohol* [28], such as taxation, will have little effect on the consumption of spirits in this region of the world. With the legal production and sale of untaxed, unrecorded spirits there is a readily available substitute if customers find taxes on recorded spirits too high. It is unlikely that informal alcohol production could be brought under formal controls, since, at present, there is in China no single agency to do this [29]. Ending the production of unrecorded bai jiu could cause economic hardship for the rural people who rely on income from this artisanal industry. And, as suggested here, unrecorded bai jiu use is somewhat regulated by custom and tradition. Under these circumstances, WHO’s caution to develop policies for unrecorded alcohol that fit the local economic and cultural context should be noted [28].

Additionally, there is evidence that some proportion of unrecorded bai jiu drinkers would prefer recorded bai jiu if given the opportunity, so unrecorded bai jiu drinking rates may decline as recorded bai jiu becomes more available and more accepted and as the older male population declines.

## 5. Conclusions

This study compared, in detail, the patterns of use of recorded and unrecorded spirits among people living in rural Central China. As such, these results add to the understanding of unrecorded alcohol in China.

The most important conclusion, at this stage, is that unrecorded alcohol is widely consumed in rural parts of China, and deserves to be examined carefully for variations and differences in the role it plays in society and in burden of disease. Only then can coherent policies to reduce public health risk and maintain public health be developed.

### Limitations

This paper was based on interviews with 3268 Chinese adults from the rural areas of three provinces in China. As such, the accuracy of the data depended upon the comprehension and honesty of the respondents. The results had significant response process validity because the data collectors were trained to probe to ensure accurate understanding of the questions and understanding the answers given. The sample does not truly represent China or even the provinces sampled or the rural populations, but it does present the first description of the users of two types of the most common alcohol consumed in rural China. The results will have considerable value for those conceptualizing more extensive studies in the future and those considering policy options to reduce risks from all types of alcohol.

## Figures and Tables

**Table 1:** Demographic characteristics of unrecorded and recorded bai jiu drinkers.

	Unrecorded Bai Jiu Drinkers		Recorded Bai Jiu Drinkers			
	N	%	N	%	χ^2^	*p*
**Gender**						
Male	1493	57.7	1094	44.3	10.3	<0.001
Female	229	49.7	232	50.3		
**Age—Males**						
<30	87	34.8	163	65.2	173.5	<0.001
30–39	162	40.7	236	59.3		
40–49	382	55.9	302	44.1		
50–59	398	64.3	221	35.7		
60+	464	73.0	172	27.0		
**Age—Females**						
<30	17	51.5	16	48.5	2.8	0.6
30–39	38	51.4	36	48.7		
40–49	59	43.7	76	56.3		
50–59	63	52.5	57	47.5		
60+	52	52.5	47	47.5		

**Table 2:** Drinking frequency of unrecorded and recorded bai jiu drinkers.

	Unrecorded Bai Jiu Drinkers		Recorded Bai Jiu Drinkers			
	N	%	N	%	χ^2^	*p*
**Drinking frequency**						
Every day	689	40.1	304	22.9	225.7	<0.001
3–6 days/week	550	31.9	297	22.4		
<3 days/week	483	28.1	725	54.7		
**Strength of spirits on last drinking occasion**						
≥42% alcohol by volume	1263	75.7	576	46.5	261.4	<0.001
<42% alcohol by volume	405	24.3	663	53.5		

**Table 3:** Estimated quantity consumed per occasion.

	Unrecorded Bai Jiu Drinkers	Recorded Bai Jiu Drinkers
Usual drinking occasions	2.7 liang	2.9 liang
Last drinking occasion	2.8 liang	2.8 liang
Most memorable drinking occasion	3.4 liang	4.6 liang

Note: one liang is approximately equal to 50 g (about 1.7 oz, and about 50 mL).

**Table 4:** Preference for recorded or unrecorded bai jiu.

	Unrecorded Bai Jiu Drinkers		Recorded Bai Jiu Drinkers	
	N	%	N	%
**Consumed their usual beverage…**				
On the last drinking occasion	1348	78.3	1236	93.2
On the most memorable occasion	1146	69.0	1172	94.4
**Which beverage would prefer if given a choice…** ^a^				
Unrecorded bai jiu	850	66.0	27	2.7
Recorded bai jiu	439	34.1	964	97.3

^a^ χ^2^ = 947.0, *p* < 0.0001.

**Table 5:** Drinking patterns—drinking pressures, heavy drinking.

	Unrecorded Bai Jiu Drinkers		Recorded Bai Jiu Drinkers			
	N	%	N	%	χ^2^	*p*
**Pressure to drink**						
Ever was pressured	877	50.9	635	47.9	2.8	0.1
Ever pressured someone else	649	37.7	480	36.2	0.7	0.4
**Heavy drinking**						
Ever felt sick/vomited after drinking on memorable occasion	494	29.7	562	45.3	74.05	<0.001
